# Diabetes Alters microRNA Expression in Epicardial and Subcutaneous Adipose Tissue from Patients Undergoing Elective Cardiac Surgery

**DOI:** 10.3390/cells15020122

**Published:** 2026-01-09

**Authors:** Diana Santos, António Canotilho, Gonçalo Coutinho, David Prieto, Pedro Antunes, Manuel Antunes, Adelino F. Leite Moreira, Inês Falcão-Pires, Eugenia Carvalho, Louise Torp Dalgaard

**Affiliations:** 1 University of Coimbra, Institute for Interdisciplinary Research, PhD Programme in Experimental Biology and Biomedicine (PDBEB), 3030-7893 Coimbra, Portugal; dfsantos@cnc.uc.pt; 2 CNC-UC—Centre for Neuroscience and Cell Biology, University of Coimbra, 3004-504 Coimbra, Portugal; 3 CiBB—Centre for Innovative Biomedicine and Biotechnology, University of Coimbra, 3004-504 Coimbra, Portugal; 4 Institute for Interdisciplinary Research, University of Coimbra, 3030-7893 Coimbra, Portugal; 5 Department of Science and Environment, Roskilde University, DK 4000 Roskilde, Denmark; 6 Cardiothoracic Surgery Unit, University Hospital of Coimbra, 3004-561 Coimbra, Portugal8058@ulscoimbra.min-saude.pt (G.C.);; 7 University Clinic for Cardiothoracic Surgery, Faculty of Medicine, University Hospital of Coimbra, 3000-548 Coimbra, Portugal; 8 UnIC@RISE, Department of Surgery and Physiology, Faculty of Medicine, University of Porto, 4200-319 Porto, Portugal

**Keywords:** epicardial adipose tissue, subcutaneous adipose tissue, microRNAs, diabetes mellitus, cardiovascular diseases

## Abstract

**Highlights:**

**What are the main findings**

- A microRNA discovery panel identified 34 microRNAs to be uniquely expressed in epicardial adipose tissue (EAT).
- Diabetes increases the expression of miR-155-5p in EAT and of miR-93-3p and miR-223-3p in subcutaneous AT.

**What are the implications of the main findings?**

- EAT microRNA targets are enriched in pathways for heart cell growth and differentiation.

**Abstract:**

Epicardial adipose tissue (EAT) function may influence the heart, given its metabolic actions and proximity to the heart. We hypothesized that diabetes mellitus (DM) alters miRNA expression across adipose tissue types, and that modifications in EAT may have critical implications for cardiac physiology. To test this, we compared EAT and subcutaneous adipose tissue (SAT) miRNA profiles between patients with and without DM and across tissues within each disease group. Paired biopsies from patients with (*n* = 18) and without DM (*n* = 46) undergoing cardiac surgery were analyzed using miRNA profiling and bioinformatics. Among 680 miRNAs screened, 34 were uniquely expressed in EAT, confirming a distinct molecular signature in this fat depot. Notably, miR-155-5p was significantly elevated in EAT from patients with DM, indicating a localized metabolic effect. In SAT, miR-93-3p and miR-223-3p were upregulated in patients with DM and consistently higher than in EAT, regardless of DM status, indicating tissue-specific regulation. miR-324-5p was more expressed in SAT of patients in the NDM group, reflecting combined effects of tissue type and DM. These patterns remained consistent across cardiac disease stratifications. Pathway analysis revealed that miRNAs enriched in EAT target genes involved in cardiomyocyte growth and differentiation. Overall, the findings highlight the unique miRNA profile of epicardial fat and its altered response to DM, supporting its relevance in cardiac physiology.

## 1. Introduction

Epicardial adipose tissue (EAT), a subtype of thoracic vascular adipose tissue, has been related to cardiac physiology modulation [1,2]. EAT is located around the coronary arteries, the visceral pericardium, and the myocardium [3]. Since there are no physical barriers between these structures, a direct crosstalk can occur between EAT cells and the cardiac milieu [4]. Despite being characterized as white adipose tissue (WAT), EAT displays brown adipose tissue (BAT)-like features due to the increased expression of mitochondrial and lipolytic proteins [5,6]. Impaired glucose regulation, hypertension, dyslipidemia, obesity, and type 2 diabetes mellitus (DM) are well-known cardiometabolic risk factors for the development of cardiovascular diseases (CVDs). These risk factors have been related to altered expression of BAT-related markers, including adipokines and microRNAs (miRNAs), as well as increased lipolytic activity and an altered secretory profile [7,8,9].

MiRNAs are small noncoding RNAs that regulate gene expression at the transcriptional level by degrading their mRNAs and/or inhibiting their translation, thereby modulating biological processes [10]. Previous studies have debated the role of miRNAs in the development of diabetic cardiovascular complications [11,12]. In fact, adipose tissue miRNAs can directly modulate the cardiovascular system [13]. However, little is still known regarding specific EAT miRNAs [4,13], and even less is known regarding their mechanisms of action or their regulation by DM.

We hypothesized that DM modifies miRNA expression in adipose tissue types, with changes in EAT having distinct implications for cardiac physiology. To address this, we compared miRNA expression in EAT and subcutaneous adipose tissue (SAT) between patients with and without DM and across tissues within each disease group. This design allowed us to characterize the miRNA profile of EAT under CVD conditions and to uncover biological processes that may be affected by DM.

## 2. Materials and Methods

### 2.1. Ethical Approval

Study participants were recruited in collaboration with the Cardiothoracic Surgery Unit at *Unidade de Saúde Local de Coimbra*, under the oversight of their Ethics Committee (approvals HUC-35-11, granted on 25 June 2012, and OBS.SF.24-2021, granted on 7 July 2021), and with the Department of Cardiothoracic Surgery at *Unidade de Saúde Local de São João*, under the approval of its Ethics Committee (CARDIAC project 109/2020, granted on 20 May 2020) [14,15,16,17]. All participants provided informed oral and written consent, and the study was conducted in accordance with the Declaration of Helsinki II.

### 2.2. Adipose Tissue Donors

This study was designed as a discovery-phase investigation with validation. The sample size (*n* = 64, 35 male and 29 female) reflects the available patient cohort undergoing elective cardiac surgery during the study period, with the primary aim of hypothesis generation rather than confirmatory statistical power. Accordingly, participants with a well-defined set of preoperative variables, including assessments of key risk factors, such as dyslipidemia, hypertension, smoking habits, and family history of cardiac disease, were recruited in collaboration with the Cardiothoracic Surgery Unit at *Unidade de Saúde Local de Coimbra* and the Department of Cardiothoracic Surgery at *Unidade de Saúde Local de São João*, [14,15,16,17]. Paired EAT and SAT biopsies (*n* = 64) were collected from patients undergoing elective coronary artery bypass grafting (CABG), valve repair, or replacement or patients undergoing combined procedures. EAT was collected from the vicinity of proximal right coronary artery and SAT from the sternum region [14,15]. To be eligible, individuals were required to be ≥18 years of age and to provide written informed consent after a comprehensive explanation of the project by the medical team. Exclusion criteria included renal impairment (serum creatinine ≥ 2 mg/dL or requirement for hemodialysis), presence of cancer or other metabolic conditions, and comorbidities significantly reducing life expectancy or affecting inflammatory responses, such as neurodegenerative diseases.

Patients were stratified for the presence or absence of DM and by the type of cardiac disease. All patients elected, at least, for a CABG were considered as the coronary artery disease (CAD) group, while all patients undergoing surgery for valve repair or replacement only were considered as the NCAD group (Figure S1). Internal controls were defined through stratification of samples by tissue type and clinical status. Specifically, SAT served as the reference tissue for EAT comparisons, while NDM patients served as the reference group for DM comparisons, and NCAD patients served as the reference group for CAD comparisons. Intrinsic demographic and clinical characteristics (age, sex, hypertension, dyslipidemia, obesity, smoking habits, and family history) were systematically recorded (see Section 3) and incorporated into stratification analyses, ensuring appropriate internal comparators and minimizing bias for meaningful interpretation.

### 2.3. MicroRNA Analysis

#### 2.3.1. RNA Isolation

After collection, adipose tissue biopsies were either snap-frozen in dry ice or placed directly into tubes containing RNAlater^®^ (Invitrogen, Carlsbad, CA, USA), for RNA stabilization, and then immediately frozen in dry ice. All biopsies were stored at −80 °C until further use. Total RNA was extracted from AT biopsies (50–200 mg) after homogenization in 1 mL TRI Reagent^®^ (Sigma Aldrich, St. Louis, MO, USA) with a TissueLyser (Qiagen, Germantown, MD, USA) following the protocol of the manufacturer. RNA integrity and concentration were assessed through Optical Density at 260/280 nm using the NanoDrop^®^ One Spectrophotometer (Wilmington, DE, USA). Diethyl pyrocarbonate (DEPC^®^, Sigma Aldrich, St. Louis, MO, USA)-treated miliQH_2_O (RNAse-free water) was used as a blank for NanoDrop calibration.

#### 2.3.2. Participant Selection for miRNA Discovery

From the whole cohort (*n* = 64), a representative subset of 32 participants (16 NDM and 16 DM) was selected for miRNA discovery. Participants were matched based on sex, age, BMI, and CAD status to minimize clinical and demographic confounding. Therefore, each group of 16 patients (NDM or DM) included 8 patients with CAD and 8 NCAD patients, as illustrated in Figure S2.

#### 2.3.3. Microarray Discovery Analysis of miRNAs

More than 680 microRNAs were profiled using two TaqMan^®^ Array platforms (Applied Biosystems, Thermo Fisher Scientific, Carlsbad, CA, USA): MicroRNA Card A v.2 (reference: 4398977) and MicroRNA Card B v.3 (reference: 4455449). Paired EAT and SAT samples from the discovery cohort were pooled into four RNA groups—Pool 1: EAT NDM; Pool 2: EAT DM; Pool 3: SAT NDM; and Pool 4: SAT DM—as shown in Figure S2. Messenger RNA from each pool was converted to cDNA by reverse transcription using the respective Megaplex^®^ RT (Applied Biosystems, Thermo Fisher Scientific, Carlsbad, CA, USA) primers required for either array type (reference: 4399966 or reference: 4444292). cDNA was then synthesized using MultiScribe Reverse Transcriptase^®^ (Applied Biosystems, Thermo Fisher Scientific, Carlsbad, CA, USA) (reference: 4366596) and added to a GeneAmp^®^ PCR 9700 System (Applied Biosystems, Thermo Fisher Scientific, Carlsbad, CA, USA). For each miRNA array, a mix containing TaqMan^®^ Universal PCR Master Mix, no Amperase UNG (reference: 4440040), was added to the specific previously prepared primer pool. The TaqMan^®^ miRNA Array cards were amplified in the VIIA 7 qPCR instrument (Applied Biosystems, Thermo Fisher Scientific, Carlsbad, CA, USA).

Normalization was performed against the global mean of Ct values for all miRNAs detected in each group, and relative expression was calculated using the ∆∆ Ct method (2^−ΔΔCt^). Only the miRNAs with a Ct value under 38 were considered for further analysis. All reagents were purchased from ThermoFisher Scientific (Thermo Fisher Scientific, Carlsbad, CA, USA).

#### 2.3.4. Validation and Targeted Analysis of Selected miRNAs

For validation, cDNA was prepared from 100 ng of total RNA using the TaqMan^®^ MultiScribe Reverse Transcription^®^ Kit (Applied Biosystems, Thermo Fisher Scientific, Carlsbad, CA, USA) (reference: 4366596) and enzyme in accordance with the manufacturer’s protocol. An exogenous cel-miRNA-39 RNA spike-in was added as a control for the reverse-transcription efficiency and used as part of normalization. cDNA synthesis was performed using the GeneAmp^®^ PCR 9700 System (Applied Biosystems, Thermo Fisher Scientific, Carlsbad, CA, USA). Primers were designed using miRNA sequence available from miRBase (version 22) at http://www.mirbase.org/ (assessed on 29 June 2022) following the procedure previously described [18,19].

The RT-qPCR amplification reactions were performed in duplicate to ensure reproducibility, using a PowerUp^®^ SYBR^®^ Green Master Mix (Applied Biosystems, Thermo Fisher Scientific, Carlsbad, CA, USA) (reference: 100029285) and 10-fold diluted cDNA in a ThermoCycler96^®^ (Roche Diagnostics, Manheim, Germany) instrument according to the manufacturer’s instructions. Expression levels were quantified using standard curve methodology, and normalized to the geometric mean of the endogenous small non-coded RNA U6 and the exogenous spike-in cel-miR-39. Synthetic RNA and oligodeoxynucleotides were purchased from TAG Copenhagen A/S, Copenhagen, Denmark, and are described in Table S1. All other reagents were purchased from ThermoFisher Scientific.

### 2.4. Statistical Analysis

Normality of continuous variables (clinical, anthropometric, and miRNA expression levels) was assessed using the Shapiro–Wilk test and Levene’s test to check variance homogeneity. For normally distributed continuous variables, comparisons between groups were performed using an unpaired Student’s *t*-test and data were presented as means ± standard errors of the mean (SEMs). Clinical and anthropometric variables, as well as miRNA expression levels, were summarized as means ± standard errors of the mean (SEMs). Categorical variables were summarized as absolute counts and percentages within each group and compared using the chi-square test. All descriptive statistics and these initial analyses were performed in SPSS software (version 28), with a significance threshold of *p* < 0.05.

For the miRNA dataset, raw values were log_2_-transformed prior to statistical testing to approximate. Heatmaps of relevant miRNAs (including the seven selected for validation) were generated in R (v. 1.3.1093, RStudio, PBC Boston, MA, USA, http://www.rstudio.com, accessed on 24 January 2024) (pheatmap package); hierarchical clustering was applied to rows using Euclidean distance and complete linkage method.

Differential expression analyses were conducted using a two-way ANOVA to evaluate the tissue type (EAT or SAT), diabetes status (NDM or DM), and even cardiac disease status. Post hoc pairwise comparisons were performed using Tukey’s multiple comparison test to control for type I error. Cross-comparisons across unrelated groups (e.g., SAT NDM vs. EAT DM) were not considered.

Linear mixed-effects models (R packages “lme4”, “lmerTest”, and “emmeans”, v. 1.3.1093, RStudio, PBC Boston, MA, USA, http://www.rstudio.com, accessed on 3 July 2025) were applied to assess the association between miRNA expression and covariates (sex and age) while accounting for tissue depot and disease status. Post hoc comparisons were performed using Tukey’s test. Results are reported as estimates (standard error).

Predicted target genes for the individual miRNAs were identified using TargetScan (v.7.2, human, http://www.targetscan.org/vert_72/, accessed on the 28 June 2024) [20].

Pathway enrichment analysis was performed using the PANTHER (Protein ANalysis THrough Evolutionary Relationships) Classification System (v.15.0, http://pantherdb.org/, accessed on 4 July 2024), as well as the PANTHER pathways annotation set. The enrichment analysis was visualized by RStudio (v. 1.3.1093, RStudio, PBC Boston, MA, USA, http://www.rstudio.com, accessed on 9 August 2024) using the ggplot2 (v.3.1.0, https://ggplot2.tidyverse.org/ accessed on 9 August 2024) package [21].

Graphical representations were prepared in GraphPad Prism version 8 (GraphPad Inc., La Jolla, CA, USA) and RStudio (v.1.3.1093, RStudio, PBC Boston, MA, USA, http://www.rstudio.com, accessed on 3 July 2025).

## 3. Results

### 3.1. Characteristics of the Study Population

**Figure 1:**
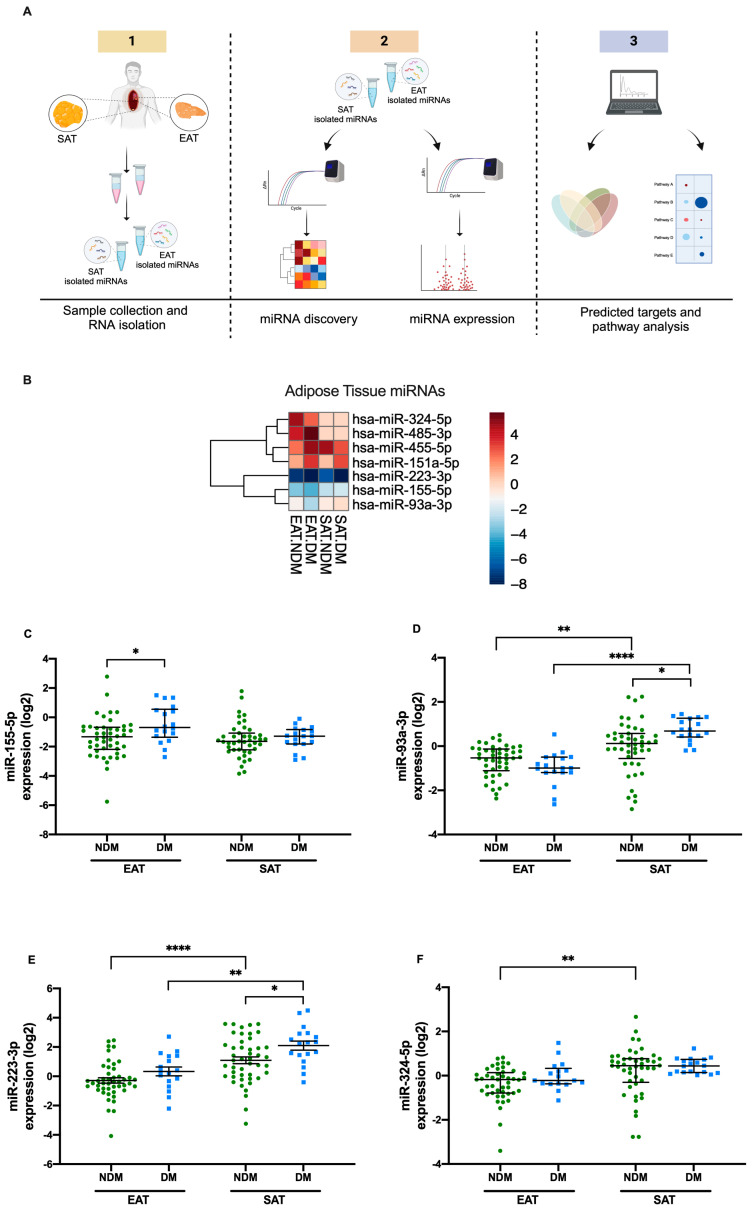
MiRNA expression levels are influenced by adipose tissue type and diabetes. (**A**) Workflow of the study; (**B**) heatmap of the selected miRNAs based on Taqman miRNA arrays; (**C**) miR-155-5p; (**D**) miR-93-3p; (**E**) miR-223-3p; and (**F**) miR-324-5p. The *p*-values were determined using a two-way ANOVA after logarithmic (log_2_) transformation. Data are presented as mean ± SEM. * *p* ≤ 0.05, ** *p* ≤ 0.001, **** *p* ≤ 0.00001. EAT, epicardial adipose tissue; DM; diabetes mellitus group; NDM, non-diabetes mellitus group; SAT, subcutaneous adipose tissue. Panel (**A**) was created in BioRender. Santos, D. (2025) https://BioRender.com/vc4a0o4, 18 November 2025.

Sixty-four patients undergoing elective cardiac surgery were recruited for this study. Of these, 46 patients without DM were assigned to the non-diabetic group (NDM), and 18 patients with type 2 DM were classified as the diabetic group (DM) (Figure 1A).

No differences were observed in the continuous variable (age and BMI) when compared using unpaired Student’s *t*-test. However, regarding the categorical variables, although no differences were found in sex distribution or in the incidence of most of the cardiovascular risk factors, hypertension differed significantly (*p* = 0.008), where 100% of the patients in the DM group had hypertension against 70% of the patients in the NDM group (Table 1). Insulin (*p* ≤ 0.001) and oral antidiabetics, such as biguanide (*p* ≤ 0.001) or a dipeptidyl peptidase-4 (DPP4) inhibitor combined with biguanide (*p* = 0.009), were only administered to the DM group. The intake of diuretic medication reached statistical significance (*p* = 0.041), with more patients being treated in the NDM group than in the DM group (Table 1). Due to the heterogeneity of the cardiac surgeries, demographic and clinical characteristics were also described according to the cardiac pathology (NCAD or CAD), as shown in Table S2. Briefly, among the 38 patients in the NCAD group, 10 had a prior diagnosis of DM, compared to 8 out of 26 patients in the CAD group. Notably, the only significant differences were found in medication usage, since 54% of patients with CAD received antiplatelet therapy (*p* = 0.05, against 24% in the NCAD group), and 50% were treated with β blockers (*p* = 0.01, against 24% in the NCAD group). Furthermore, the presence of DM did not significantly influence cardiac pathology manifestation in either NCAD or CAD groups, as described in Table S2.

### 3.2. miRNA Discovery: Cohort Description and Adipose Tissue-Dependent Profiling

Clinical and demographic characteristics of the discovery cohort (*n* = 16 NDM vs. *n* = 16 DM) are summarized in Table S3. They closely reflect those of the full study population, supporting the representativeness of this subset for miRNA profiling (Figure S2 and Table S3).

Of the 680 miRNAs included in the array, 146 were commonly expressed across the four study conditions (EAT NDM, EAT DM, SAT NDM, and SAT DM). In EAT, 254 miRNAs were detected in the NDM group and 229 in the DM group. Regarding SAT, 186 miRNAs were detected in the NDM group, and 203 miRNAs were detected in the DM group. Importantly, 34 miRNAs were exclusively expressed in EAT, while none were found to be unique to SAT. To further illustrate these differential expression patterns, a heatmap of the discovery cohort is provided in the Supplementary Material (Figure S3), with distinct color coding for the four study conditions (EAT NDM, EAT DM, SAT NDM, SAT DM). In this representation, dark red indicates higher relative expression, whereas dark blue denotes lower relative expression.

Candidate miRNAs for further analyses were selected based on the following criteria: (a) those showing the greatest differential expression between adipose tissue depots independent of DM status (EAT vs. SAT); (b) those with the largest differential expression between adipose tissues when stratified by DM status (NDM: EAT vs. SAT; DM: EAT vs. SAT); (c) those most differentially expressed within each tissue type when comparing patients with and without DM (EAT: NDM vs. DM; SAT: NDM vs. DM), as the most relevant miRNAs, are presented in Figure S3.

Thus, based on the screening results and the supporting literature (see Table S4), seven miRNAs were selected for individual validation by RT-qPCR in the full cohort of 64 participants. These miRNAs were prioritized because of their established roles in biological processes directly relevant to our hypothesis, including inflammation (miR-155-5p [22] and miR-223-3p [23,24,25,26,27,28]), metabolic regulation and adipogenesis (miR-93-3p [29,30,31,32,33,34], miR-324-5p [35,36,37,38], and miR-151a-5p [39,40]), and cardiovascular pathology (miR-485-3p [41,42,43] and miR-455-5p [4,44,45]). Their biological relevance is summarized in Table S4.

### 3.3. Altered miRNA Expression in EAT and SAT from Patients Elected for Cardiac Surgery

To evaluate whether DM or adipose tissue type influenced the expression of the selected microRNAs, the four study conditions (EAT NDM, EAT DM, SAT NDM, and SAT DM) were compared using a two-way ANOVA with Tukey’s multiple comparison test. In EAT, patients with DM exhibited increased expression of miR-155-5p (*p* = 0.043) compared to the NDM group (Figure 1C). Additionally, in SAT, increased expression levels of both miR-93-3p (*p* = 0.015) and miR-223-3p (*p* = 0.042) were observed in patients with DM relative to the NDM group (Figure 1D and Figure 1E, respectively).

Furthermore, tissue type significantly influenced the expression of miR-93-3p and miR-223-3p and miR-324-5p. In the NDM group, miR-93-3p levels (*p* = 0.006) were reduced by 50% in EAT compared to SAT (Figure 1D), while miR-223-3p expression (*p* ≤ 0.0001) was approximately three-fold lower in EAT compared to SAT (Figure 1E). Additionally, in these patients, miR-324-5p expression in EAT was nearly 50% (*p* = 0.009) lower than in SAT (Figure 1F). Similarly, in the DM group, expression levels of miR-93-3p (*p* ≤ 0.0001) and miR-223-3p (*p* = 0.001) were reduced by approximately 30% in EAT when compared to SAT (Figure 1D and Figure 1E, respectively).

However, no differences in the expression levels of miR-151a-5p, miR-455-5p, or miR-485-3p were observed between tissues or disease status (Figure S4A–C).

Given the heterogeneity of cardiac surgeries, we further evaluated whether the type of cardiac disease could influence the expression of the selected miRNAs. For this analysis, the cohort was stratified according to cardiac disease and surgery, resulting in four study conditions—EAT NCAD, EAT CAD, SAT NCAD, and SAT CAD—and was compared using a two-way ANOVA with Tukey’s multiple comparison test. No significant differences in miRNA expression were observed regarding cardiac disease type for either EAT or SAT. However, differences emerged when comparing tissue types (Figure S5).

In the NCAD group, expression levels of miR-93-3p (*p* = 0.0007), miR-223-3p (*p* = 0.0002), and miR-324-5p (*p* = 0.03) were significantly decreased in EAT compared to SAT (Figure S5A–C). Similarly, in the CAD group, miR-93-3p (*p* = 0.0004) and miR-223-3p (*p* = 0.001) also showed decreased expression in EAT compared to SAT (Figure S5A and S5B, respectively). No significant differences in expression levels were detected for miR-151a-5p, miR-455-5p, or miR-485-3p across tissues or cardiac disease types (Figure S6A–D).

### 3.4. Interaction Between miRNAS and Anthropometric Disease Status Variables

Analysis using linear mixed-methods models showed a significant effect of tissue type on the expression of miR-93a-3p (*p* ≤ 0.001), miR-223-3p (*p* ≤ 0.001), miR-324-5p (*p* ≤ 0.001), and miR-151a-5p (*p* = 0.009); all showed higher expression in SAT relative to EAT. The expression of miR-155-5p was significantly increased by DM status (*p* = 0.02). A significant effect of sex was observed on the expression levels of miR-485-3p (*p* = 0.02), with higher expression in female individuals, as described in the top section of Table 2.

Furthermore, the final two rows of Table 2 report the effect of DM within each type of tissue when adjusted for sex and age. Thus, the expression of miR-155-5p remained significantly associated with DM in EAT (*p* = 0.02). In contrast, in SAT, both miR-93a-3p (*p* ≤ 0.001) and miR-223-3p (*p* ≤ 0.001) were associated with DM, as detailed in Table 2.

Similarly, we evaluated the association between the same variables in miRNA expression levels, tissue type, sex and age, and the presence of CAD, as detailed in Table S5. According to the linear mixed-effects model, the expression levels of miR-155-5p (*p* = 0.03), miR-93a-3p (*p* ≤ 0.001), miR-223-3p (*p* ≤ 0.001), miR-324-5p (*p* ≤ 0.001), and miR-151a-5p (*p* < 0.01) were significantly influenced by tissue type, all showing a higher expression in SAT. The expression levels of miR-324-5p (*p* < 0.05) were also affected by age. Moreover, after adjustment for anthropometric variables, no significant effect of CAD was observed on miRNA expression levels within each type of adipose tissue (Table S5).

To provide an integrated overview of these findings, dedicated summary tables were compiled. Table 3 consolidates the effect of DM status (DM vs. NDM) and adipose tissue (EAT vs. SAT) on miRNA expression, incorporating results from both ANOVA/Tukey and linear mixed effects-models.

The corresponding analysis for CAD status, performed under the same statistical framework and variable structure, is presented in Table S6.

### 3.5. Pathway Analysis

Altered miRNA expression levels can directly affect the expression of their predicted targets and, consequently, influence metabolic pathway activity. For the four miRNAs (miR-155-5p, miR-93-3p, miR-223-3p, and miR-324-5p) consistently altered by either tissue type and/or the presence of DM, there was a total of 5524 predicted target genes.

However, to interrogate the adipose tissue specificities of the pathway analysis, the predicted target genes were filtered according to mRNAs that are expressed in EAT and SAT based on the combination of the data from the GSE108971 and GSE179455 transcriptome expression datasets [from the Gene expression Omnibus (GEO) database], in order to limit the evaluation of the predicted targets to those with relevance for these specific tissues.

**Figure 2:**
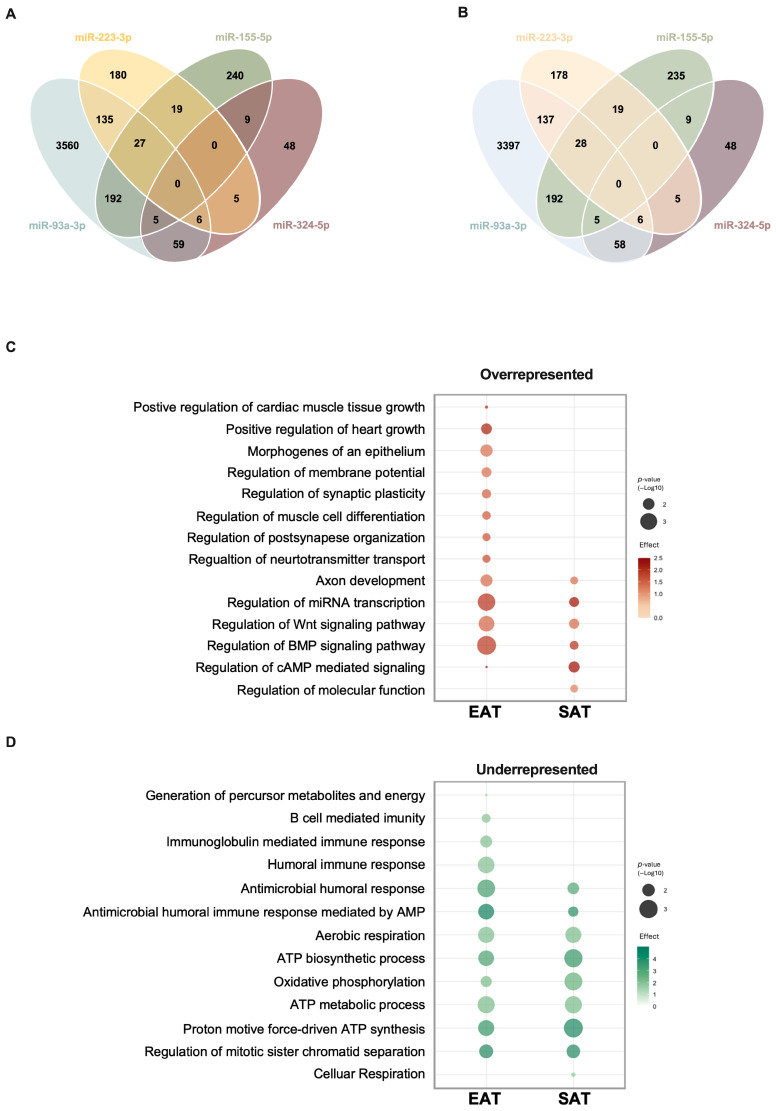
Predicted target genes and pathway analysis. Venn diagram representing the number of individual and shared target genes of the differentially expressed miRNAs for (**A**) EAT and for (**B**) SAT. The enrichment analysis of the (**C**) overrepresented and the (**D**) underrepresented pathways regulated by the differentially expressed miRNAs. AMP, antimicrobial peptide; ATP, adenosine triphosphate; BMP, bone morphogenic protein; cAMP, cyclic adenosine monophosphate; EAT, epicardial adipose tissue; SAT, subcutaneous adipose tissue; Wnt, wingless-related integration site.

In EAT, a total of 4980 predicted transcripts were identified as predicted targets. Among these, 80% (3982) were targets of miR-93-3p, while 492 were targeted by miR-155-5p, 372 by miR-223-3p, and only 132 predicted transcripts were predicted to be targeted by miR-324-5p (Figure 2A). For SAT, a total of 3858 predicted targets were identified. Here again, most of the identified transcripts (3823) were predicted targets of miR-93-3p, followed by miR-155-5p and by miR-223-3p, with 488 and 373 predicted targets, respectively, and only 131 predicted transcripts were identified as predicted targets of miR-324-5p (Figure 2B).

Most of the predicted targets for each miRNA were expressed in both adipose tissue types. However, for miR-93-3p, 220 transcripts were only identified as predicted targets in EAT, while 59 predicted targets were only observed in SAT. For miR-155-5p, 15 and 11 targets were only identified in EAT and SAT, respectively. Moreover, for EAT, four predicted transcripts were uniquely observed as predicted targets either for miR-223-3p or miR-324-5p, while for SAT, five and four predicted targets were identified, respectively (Table S7).

There was no overlap in the predicted targets when all four miRNAs were combined, meaning that no single mRNA is targeted by all four regulated miRNAs (Figure 2A). However, several predicted targets are shared by the combination of other two or more miRNAs. Therefore, for the pathway analysis study, a combined set of predicted targets with relevance to either EAT or SAT was used. Interestingly, both EAT and SAT shared a total of 167 pathways, while 49 pathways were identified as specific to EAT and only 2 pathways were identified exclusively for SAT (Table S8).

Based on the pathway enrichment analysis performed with Panther, we conducted a gene ontology analysis focused on biological processes (BPs) for the combined list of predicted target mRNAs of the four regulated miRNAs. The presentation of these was divided into those that were depleted for the annotation terms targeted by these miRNAs (Figure 2C) and those that were enriched (Figure 2D).

Several pathways were overrepresented among predicted mRNA targets, either in EAT or in SAT. In EAT, an overrepresentation of several positive regulation pathways related to tissue and organ growth was observed, with “Positive regulation of the heart growth” (*p* = 0.013) having the highest enrichment of 2.1-fold, followed by “Positive regulation of cardiac muscle tissue growth”, with an enrichment of 2.04-fold (*p* ≤ 0.05) (Figure 2C). In addition, several neural innervation-related pathways were also overrepresented in EAT. These pathways included “Regulation of neurotransmitter transport” (enrichment fold of 1.64, *p* = 0.026) and “Regulation of post synapse organization” (enrichment fold of 1.58, *p* = 0.027) (Figure 2C). Interestingly, with an enrichment of 1.13-fold, “Regulation of molecular function”, a very broad pathway, was the only overrepresented pathway observed only in SAT (*p* = 0.028) (Figure 2C). Nonetheless, both adipose tissue types had an overrepresentation of the “Regulation of cAMP-mediated signaling” pathway, with a 2.04-fold enrichment (*p* ≤ 0.05) for EAT and 2.26-fold (*p* = 0.012) for SAT, followed by “Regulation of miRNA transcription”, with an enrichment of 1.89-fold (*p* = 0.0007) for EAT and an enrichment of 2.22-fold (*p* = 0.017) for SAT (Figure 2C).

Several underrepresented pathways, to which fewer targeted mRNAs were annotated than expected by chance, were common to both adipose tissue types. These are pathways which in those tissues appear less targeted by the regulated miRNAs. However, there were significantly depleted pathways that were EAT-specific, including the immune response-related pathways “B-cell mediated immunity” (fold depletion of −0.54, *p* = 0.025) and “Immunoglobulin mediated immune response” (fold depletion of −0.51, *p* = 0.012). The “Generation of precursor metabolites and energy” pathway was also significantly depleted in EAT (−0.74-fold, *p* = 0.05) (Figure 2D). With a fold depletion of −0.61 (*p* = 0.05), the only underrepresented pathway observed specifically in SAT was that of “Cellular respiration”, a general pathway that includes all the metabolic pathways related to ATP production from glucose (Figure 2D). The “Antimicrobial humoral immune response mediated by antimicrobial peptide” pathway was the most underrepresented in EAT, with a fold depletion of −0.22 (*p* = 0.003), but it was also relevant to SAT (depletion of −3.85, *p* = 0.019). Moreover, “Regulation of mitotic sister chromatid separation”, a pathway which may indicate a low proliferative and hyperplasia rate, was also underrepresented in both adipose tissue types (EAT: fold depletion of −4.17, *p* = 0.006; SAT: fold depletion of −0.26, *p* = 0.007) (Figure 2D).

## 4. Discussion

With the global rise in obesity, DM, and CVD, the interest in adipose tissue biology has intensified. This is particularly true under pathological conditions, due to a better understanding of their plasticity and adaptation to the local environment [46]. Adipose tissue morphology and function are increasingly recognized as depot-specific, with distinct characteristics depending on the anatomical location [47]. Among the adipose depots of interest, EAT, a subtype of visceral adipose tissue (VAT) with BAT-like features, is in the vicinity of the heart, in close proximity to cardiomyocytes and the surrounding cells. Multiple studies have reported critical disease-associated changes in the structural and functional characteristics of EAT, particularly in the context of DM and CAD, which have been comprehensively addressed in our previously published review [13]. Over the past three decades, miRNAs, small RNA transcripts with only ~21 nucleotides, have emerged as key post-transcriptional regulators, involved in cellular development, differentiation, maturation, and regeneration or disease progression [48,49]. In fact, EAT-derived miRNAs are increasingly viewed as promising therapeutic strategies for either prevention and/or treatment of CVD. Therefore, this study aimed to determine whether the expression profiles of selected miRNAs are influenced by adipose tissue depot, the presence of metabolic or cardiovascular disease, or both, with potential implications for adipose tissue function and inflammatory regulation. Our findings revealed distinct tissue- and disease-associated miRNA expression patterns, underscoring regulatory differences between EAT and SAT, as well as between patients with and without DM. These findings highlight critical pathways of adipose–cardiac interaction, although mechanistic studies are required to establish causality.

A total of 64 participants were included in this study. Although the NDM group had more than twice the number of participants compared with the DM group, the cohort remained largely comparable across most demographic and clinical characteristics. One exception was the prevalence of hypertension, which was significantly higher in the DM group. In fact, while 70% of the NDM group had a clinical diagnosis of hypertension, this rate reached 100% among those with DM. As expected, antidiabetic medication was exclusively used by patients with DM. Additionally, despite the greater absolute number of patients with indication for diuretic therapy in the NDM group, the proportion of patients receiving diuretics was higher in the DM group (33% NDM vs. 67% DM) when adjusted for group size.

Based on the miRNA discovery analysis, which included 16 NDM and 16 DM participants, seven miRNAs were identified as biologically relevant, either due to adipose tissue specificity, disease association, or both. These were miR-324-5p [35,36,37,38], miR-485-3p [41,42,43], miR-455-5p [4,44,45], miR-151a-5p [39,40], miR-223-3p [23,24,25,26,27,28], miR-155-5p [22], and miR-93-3p [29,30,31,32,33,34], as described in Table S4.

The results revealed a significantly altered miRNA expression in adipose tissue depots, particularly in the context of DM. miR-155-5p levels were markedly elevated in EAT from patients with DM, consistent with its reported roles in glucose uptake and inflammatory signaling, which might reflect a pro-inflammatory state within this tissue [50]. In contrast, the expression of miR-93a-3p and miR-223-3p was increased in SAT from individuals with DM, suggesting a tissue-specific metabolic response [23,29]. The distinct expression patterns of miR-93a-3p and miR-223-3p in SAT vs. EAT, independent of DM, further highlight the physiological differences and unique regulatory environments of these two adipose depots. Similarly, the expression of miR-324-5p was regulated by both tissue type and metabolic condition, with increased levels in SAT in the NDM group.

A linear mixed-effects model adjusting for differences in age and sex between patients reinforced these observations, demonstrating that DM is an independent determinant of altered miRNA expression. Specifically, DM is associated with increased levels of miR-155-5p in EAT and both miR-93a-3p and miR-223-3p in SAT. These findings support the potential of these miRNAs as biomarkers of metabolic stress contributing to the DM pathophysiological alterations occurring within these adipose tissues in DM. Importantly, tissue type emerged as a more important factor than CAD status for miR-93a-3p, miR-223-3p, miR-324-5p, and miR-151a-5p, indicating that local depot biology exerts greater influence on these particular miRNAs than cardiac disease alone.

The significant upregulation of miR-155-5p in EAT of DM patients holds important physiological implications. Given its established roles as a positive regulator of glucose uptake in insulin-sensitive cells [51] and as a key mediator of inflammation, its elevated levels may reflect a pro-inflammatory state within this cardioprotective fat depot. Upregulation of miR-155-5p has been considered a pro-inflammation signal [22,52,53]. In addition, its expression appears to be stabilized after adipocyte differentiation [50]. This suggests that miR-155-5p may contribute to EAT dysfunction, in turn negatively impacting local cardiac health.

Similarly, the consistent upregulation of miR-93a-3p and miR-223-3p in SAT of DM patients points to their significant impact on metabolic and inflammatory pathways. Both miRNAs are involved in adipocyte differentiation, with miR-93a-3p acting as a negative regulator of brown adipocyte differentiation, which is relevant due to the brown adipocyte-like characteristics of EAT [54]. Furthermore, miR-223-3p is a well-documented contributor to adipocyte dysfunction and insulin resistance [23,24], and its elevated expression is directly linked to the suppression of insulin-stimulated glucose uptake [23]. Therefore, the increased expression of these miRNAs in SAT could drive local metabolic dysregulation, contributing to the systemic effects of DM. Additionally, both miRNAs have been associated with CVD progression [25,30,31,32].

The differential expression of miR-324-5p between EAT and SAT, with lower levels in EAT in the NDM group, suggests a potential link to the cardioprotective function of EAT. This miRNA is a known regulator of lipid accumulation and inflammation [35,36,37]. Its altered expression in the context of DM may disrupt a protective regulatory mechanism, while its increased levels in SAT are associated with increased risk of the metabolic syndrome [38]. Therefore, this observation highlights how the same miRNA may have distinct tissue-specific effects with different physiological outcomes.

Important to note that, although all four miRNAs are associated with stages of adipocyte function [23,29,35,36,37,51,54], there was no overlap when their predicted transcripts were evaluated in a pathway analysis for either EAT or SAT expressed transcripts. Even though the core of predicted targets was similar in both types of adipose tissue, we identified three times higher numbers of specific predicted targets in EAT than in SAT (243 vs. 79). These results influence the predicted pathway analysis and accentuate the differences between these adipose tissue types [47,55]. Notably, a quarter of the pathways identified after the gene ontology analysis (biological processes, BPs) were restricted to EAT, while only two were SAT-specific. Several of these pathways were partially redundant, especially those exclusive to SAT. In fact, “Cellular respiration”, a general pathway that includes all the metabolic pathways related to glucose breakdown for ATP production (glycolysis, pyruvate oxidation, Krebs cycle, and oxidative phosphorylation), is the only underrepresented pathway exclusive to SAT. In general, pathways related to either innate or adaptative immune responses, as well as energy production and consumption, were less likely to be targeted by the regulated miRNAs, especially in EAT, but also in SAT.

The pathway analysis further supports the notion of distinct functional roles for these miRNAs in EAT and SAT. More pathways were exclusive to EAT, emphasizing its unique contribution to the cardiac milieu [56]. The results suggest that the identified miRNAs can modulate fundamentally different physiological processes in these tissues. “Regulation of cAMP-mediated signaling” was the only pathway overrepresented in both tissues, which is consistent with the key role of cAMP in adipocyte differentiation [57] and browning [58].

This study has several limitations that deserve consideration. Consistent with our previous reports [14,15], the most significant limitation is the absence of a truly healthy control group. Given the ethical constraints of collecting EAT only during cardiac surgery, our NDM and NCAD cohorts were used as internal references. However, their underlying cardiac indications likely reflect subclinical pathologies, which limits the interpretation of a true healthy baseline metabolic profile. This is particularly relevant as insulin resistance and other metabolic syndrome-related comorbidities have been described in cardiovascular disease, even in patients without a formal diagnosis of DM [59]. A second key limitation is that our study cohort was not medication-naïve. Patients were receiving various therapies, including drugs with hypoglycemic effects for those with DM. Additionally, other factors such as sex, age, BMI, and disease severity are known to influence miRNA expression. Although these variables did not show any significant differences when groups were stratified by diabetes (Table 1) or CAD status (Table S2), exploratory analyses revealed a sex-specific effect on miR-485-5p in SAT, with higher expression observed in females (Table S9). However, for the specific miRNAs investigated in the current study, the data did not reveal any associations between age and miRNA abundance.

Finally, the last limitation is related to the study design, since it focused on the validation of a selected panel of miRNAs and used a cross-sectional design, which precludes conclusions about temporal changes or causality. Overall, while these studies reflect a real-world clinical setting and enhance the translational relevance of the findings, pharmacological treatments and patient heterogeneity may have influenced adipose tissue metabolism, potentially obscuring some disease-specific differences.

## 5. Conclusions

This study demonstrates that adipose tissue type exerts a stronger influence on miRNA expression than disease status alone, with SAT showing more pronounced alterations than EAT. This may be of relevance, since SAT has been considered a metabolically protective tissue type [60]. The data also suggest that DM, rather than CAD, is the primary clinical driver of these changes. Notably, SAT exhibited increased expression of miRNAs with established roles in pro-inflammatory signaling and metabolic dysregulation, highlighting its heightened sensitivity to systemic metabolic stress. In contrast, the relatively muted response in EAT may reflect its distinct physiological role, including its proximity to the myocardium and its involvement in local cardiac homeostasis, indicating a more regulated or delayed adaptation to disease progression. Importantly, the strength of our conclusions is supported by a well-characterized patient cohort, the use of pairwise comparisons where feasible, and a mixed-methods analytical strategy that enhances the reliability and robustness of human data interpretation. Thus, the differential expression of miR-155-5p, miR-93-3p, miR-223-3p, and miR-324-5p underscores their potential as biomarkers and therapeutic targets for DM and CVD. Nevertheless, further mechanistic and longitudinal studies are required before clinical translation can be considered.

## Figures and Tables

**Table 1:** Demographic and clinical characteristics of the study population (*n* = 64).

	NDM	DM	*p*-Value
N	46	18	
Male (M)	25 (54%)	10 (56%)	0.93
Age (years)	70.1 ± 1.6	72.2 ± 1.5	0.34
Cardiovascular risk factors			
Hypertension	32 (70%)	18 (100%)	**0.008**
Dyslipidemia	36 (78%)	13 (72%)	0.61
Smoking	11 (24%)	4 (22%)	0.89
BMI	27.3 ± 0.5	27.0 ± 0.7	0.74
Family history of heart disease	1 (2%)	1 (6%)	0.58
Medication			
Antiplatelet	14 (30%)	9 (50%)	0.32
Antiarrhythmic	4 (9%)	4 (22%)	0.23
Anticoagulant	8 (17%)	5 (28%)	0.56
Insulin	0 (0%)	6 (33%)	**≤0.001**
Oral antidiabetic			
Biguanide	0 (0%)	8 (44%)	**≤0.001**
DPP4 inhibitor	0 (0%)	1 (6%)	0.14
DPP4 inhibitor + Biguanide	0 (0%)	3 (17%)	**0.009**
Sulfonylurea	0 (0%)	1 (6%)	0.14
Diuretic	15 (33%)	12 (67%)	**0.041**
ACEI	13 (28%)	8 (44%)	0.43
ARB	12 (26%)	5 (28%)	0.72
β blocker	13 (28%)	9 (50%)	0.23
Calcium channel blocker	6 (13%)	2 (11%)	0.64
Electrolyte—KCl	1 (2%)	1 (6%)	0.58
Statins	27 (59%)	12 (67%)	0.73

Quantitative measurements (BMI and age) are presented as means ± SEMs, and an unpaired Student’s *t*-test was performed. Categorical variables are reported as n (%) and compared using a chi square test. Significant *p*-values (*p* ≤ 0.05) are highlighted in bold. ACEI, angiotensin-converting enzyme inhibitor; ARB, angiotensin II receptor blockers; BMI, body mass index; DM; diabetes mellitus group; DPP-4, dipeptidyl peptidase-4; NDM, non-diabetes mellitus group.

**Table 2:** Linear mixed-methods model estimating the effects of type of tissue, anthropometric characteristics, and DM status on miRNA expression (*n* = 64).

Term	miR- 155-5p	miR- 93a-3p	miR- 223-3p	miR- 324-5p	miR- 151a-5p	miR- 455-5p	miR- 485-3p
Intercept	**−2.557** (0.882) **	−0.938 (0.705)	−1.023 (0.886)	**−1.726** (0.697) *	−1.395 (0.748)	**−2.002** (0.726) **	−0.833 (0.527)
DM	**0.796** (0.331) *	−0.323 (0.253)	0.502 (0.385)	0.340 (0.241)	0.297 (0.259)	0.084 (0.269)	−0.376 (0.218)
Tissue	−0.328 (0.230)	**0.650** (0.144) ***	**1.292** (0.282) ***	**0.581** (0.127) ***	**0.354** (0.132) **	0.313 (0.165)	−0.063 (0.165)
Age	0.018 (0.012)	0.003 (0.010)	0.010 (0.012)	0.019 (0.010)	0.015 (0.011)	0.016 (0.010)	0.003 (0.007)
Sex	−0.044 (0.230)	0.027 (0.193)	0.279 (0.242)	0.092 (0.187)	0.123 (0.205)	−0.018 (0.199)	**0.253** (0.140) *
DM: Tissue	−0.625 (0.427)	**1.069** (0.270) ***	0.483 (0.536)	−0.141 (0.240)	−0.065 (0.248)	−0.019 (0.310)	0.380 (0.308)
DM: EAT	**−0.796** (0.331) *	0.323 (0.253)	−0.502 (0.385)	−0.340 (0.242)	−0.297 (0.259)	−0.084 (0.270)	0.376 (0.218)
DM: SAT	−0.170 (0.332)	**−0.747** (0.253) ***	**−0.985** (0.375) ***	−0.199 (0.237)	−0.233 (0.259)	−0.064 (0.269)	−0.004 (0.218)

A linear mixed-methods model was created, and data are presented as estimates (SE). The upper section displays model estimates for each variable. The final two rows summarize the Tukey-adjusted comparisons evaluating the effect of DM status within each tissue, adjusted for age and sex. Significant *p*-values (*p* ≤ 0.05) are highlighted in bold; * *p* < 0.05. ** *p* < 0.01 and *** *p* < 0.001. DM, diabetes mellitus group; EAT, epicardial adipose tissue; SAT, epicardial adipose tissue.

**Table 3:** Summary of miRNA expression changes according to DM status in EAT and SAT.

	ANOVA		Mixed-Methods Analysis		
miRNA	Effect of Tissue	Effect of DM	Effect of Tissue	Effect of DM	Interaction (DM: Tissue)
miR- 155-5p	–	EAT: ↑ in DM vs. NDM (*p* = 0.043)	–	↑ associated with EAT (*p* = 0.02)	–
miR- 93a-3p	NDM: ↓ 50% in EAT vs. SAT (*p* = 0.006)	SAT: ↑ in DM vs. NDM (*p* = 0.015)	+ associated with SAT (*p* ≤ 0.001)	NS	+ associated with SAT (*p* ≤ 0.001)
	DM: ↓ 30% in EAT vs. SAT (*p* ≤ 0.0001)				
miR- 223-3p	NDM: ↓ ~67% in EAT vs. SAT (*p* ≤ 0.0001)	SAT: ↑ in DM vs. NDM (*p* = 0.042)	- associated with SAT (*p* ≤ 0.001)	NS	- associated with SAT (*p* ≤ 0.001)
	DM: ↓ 30% in EAT vs. SAT (*p* = 0.001)				
miR- 324-5p	NDM: ↓ ~50% in EAT vs. SAT (*p* = 0.0009)	–	+ associated with SAT (*p* ≤ 0.001)	–	–
miR- 151a-5p	–	–	+ associated with SAT (*p* = 0.009)	–	–
miR- 455-5p	–	–	–	–	–
miR- 485-3p	–	–	–	–	–

The *p*-values were determined using a two-way ANOVA after logarithmic (log_2_) transformation or by applying a linear mixed-methods model. *p* values were considered significant at *p* ≤ 0.05. ↓, decreased by; ↑, increased by; +, positively; -, negatively; EAT, epicardial adipose tissue; DM, diabetes mellitus group; NDM, non-diabetes mellitus group; SAT, epicardial adipose tissue.

## Data Availability

The data that support the findings of this study are available from the corresponding author upon reasonable request.

## Supplementary Materials

The following supporting information can be downloaded at https://www.mdpi.com/article/10.3390/cells15020122/s1. Table S1. Nucleotide sequences from the target miRNAs used in the present study. Table S2. Demographic and clinical characteristics of the study population according to cardiac disease (*n* = 64). Table S3. Demographic and clinical characteristics of the study population selected for miRNA discovery (*n* = 32). Table S4. Adipose tissue-related miRNAs with potential impact on diabetes and cardiac disease. Table S5. Linear mixed-methods model estimating the effects of tissue type, anthropometric characteristics, and CAD status on miRNA expression (*n* = 64). Table S6. Summary of miRNA expression changes according to CAD status in EAT and SAT. Table S7. Specific target gene symbols for each tissue. Table S8. Predicted gene ontology analysis (biological processes, BPs) pathways specific to each tissue. Gene ontology pathways. Table S9. Linear mixed-methods model estimating the effects of tissue type, anthropometric characteristics, disease status, and sex on miRNA expression (*n* = 64). Figure S1. Schematical representation of patient recruitment and group stratification according to the presence or absence of diabetes and cardiac disease. EAT, epicardial adipose tissue; CAD, coronary disease group; DM, diabetes mellitus group; NCAD, non-coronary disease group; NDM, non-diabetes mellitus group; SAT, subcutaneous adipose tissue. Figure S2. Schematical representation of the epicardial and subcutaneous adipose biopsies used for RNA pool. EAT, epicardial adipose tissue; CAD, coronary disease group; DM, diabetes mellitus group; NCAD, non-coronary disease group; NDM, non-diabetes mellitus group; SAT, subcutaneous adipose tissue. Figure S3. Heatmap of the most relevant miRNAs expressed in EAT and SAT. EAT, epicardial adipose tissue; DM, diabetes mellitus group; NDM, non-diabetes mellitus group; SAT, subcutaneous adipose tissue. Figure S4. MiRNAs expression levels in EAT and SAT regarding the presence or absence of DM. (a) miR-151a-5p, (b) miR-455-5p, and (c) miR-485-3p. *p*-values were determined using a two-way ANOVA after logarithmic (log_2_) transformation. Data are presented as means ± SEMs. EAT, epicardial adipose tissue; DM, diabetes mellitus group; NDM, non-diabetes mellitus group; SAT, subcutaneous adipose tissue. Figure S5. Adipose tissue miRNA levels are influenced by tissue type under the same cardiac disease. (a) miR-93a-3p, (b) miR-223-3p, (c) miR-324-5p. *p*-values were determined using a two-way ANOVA after logarithmic (log_2_) transformation. Data are presented as means ± SEMs. * *p* ≤ 0.05; *** *p* ≤ 0.0001. EAT, epicardial adipose tissue; CAD, coronary disease group; NCAD, non-coronary disease group; SAT, subcutaneous adipose tissue. Figure S6. miRNA expression levels in EAT and SAT according to cardiac disease. (a) miR-155-5p, (b) miR-151a-5p, (c) miR-455-5p, and (d) miR-485-3p. *p*-values were determined using a two-way ANOVA after logarithmic (log_2_) transformation. Data are presented as means ± SEMs. EAT, epicardial adipose tissue; CAD, coronary disease group; NCAD, non-coronary disease group; SAT, subcutaneous adipose tissue.

## Abbreviations

The following abbreviations are used in this manuscript:

ACEI	Angiotensin-converting enzyme inhibitor
ARB	Angiotensin II receptor blockers
AMP	Antimicrobial peptide
ATP	Adenosine triphosphate
BAT	Brown adipose tissue
BMI	Body mass index
BMP	Bone morphogenic protein
BP	Biological process
cAMP	Cyclic adenosine monophosphate
CABG	Coronary artery bypass grafting
CAD	Coronary artery disease/coronary artery disease group
CVD	Cardiovascular disease
DEPC	Diethyl pyrocarbonate
DM	Diabetes mellitus/diabetes mellitus group
DPP-4	Dipeptidyl peptidase-4
EAT	Epicardial adipose tissue
GEO	Gene expression omnibus
miRNA	microRNA
NCAD	Non-diabetes mellitus group
NDM	Non-coronary artery disease group
SAT	Subcutaneous adipose tissue
SEM	Stander error of the median
WAT	White adipose tissue
WNT	Wingless-related integration site
