# Supplementary material for: Diabetes Alters microRNA Expression in Epicardial and Subcutaneous Adipose Tissue from Patients Undergoing Elective Cardiac Surgery
Source: Cells. 2026 Jan 9;15(2):122. doi: 10.3390/cells15020122 (PMC12838747; doi:10.3390/cells15020122)
Supplement: Supplementary file 1 [file cells-15-00122-s001.zip › cells-3988750-supplementary.pdf]

cells

MDPI

Article

Diabetes Alters microRNA Expression in Epicardial and Subcutaneous Adipose Tissue from Patients Undergoing Elective Cardiac Surgery

Diana Santos [1,2,3,4,5], António Canotilho [6], Gonçalo Coutinho [6], David Prieto [6], Pedro Antunes [6], Manuel Antunes [7], Adelino F. Leite Moreira [8], Inês Falcão-Pires [8], Eugenia Carvalho [2,3,4,*] and Louise Torp Dalgaard [5,*]

1 University of Coimbra, Institute for Interdisciplinary Research, PhD Programme in Experimental Biology and Biomedicine (PDBEB), 3030-7893 Coimbra, Portugal; dfsantos@cnc.uc.pt
2 CNC-UC—Centre for Neuroscience and Cell Biology, University of Coimbra, 3004-504 Coimbra, Portugal
3 CiBB—Centre for Innovative Biomedicine and Biotechnology, University of Coimbra, 3004-504 Coimbra, Portugal
4 University of Coimbra, Institute for Interdisciplinary Research, 3030-7893 Coimbra, Portugal
5 Department of Science and Environment, Roskilde University, DK 4000 Roskilde, Denmark
6 Cardiothoracic Surgery Unit, University Hospital of Coimbra, 3004-561 Coimbra, Portugal; 8058@ulscoimbra.min-saude.pt (G.C.)
7 University Clinic for Cardiothoracic Surgery, Faculty of Medicine, University Hospital of Coimbra, 3000-548 Coimbra, Portugal
8 UnIC@RISE, Department of Surgery and Physiology, Faculty of Medicine, University of Porto, 4200-319 Porto, Portugal
* Correspondence: ecarvalh@cnc.uc.pt (E.C.), Tel.: +351-239820190 (E.C.); ltd@ruc.dk (L.T.D.)

Academic Editor: Raj Kishore

Received: 30 October 2025
Revised: 5 January 2026
Accepted: 7 January 2026
Published: 9 January 2026

Copyright: © 2026 by the authors. Licensee MDPI, Basel, Switzerland. This article is an open access article distributed under the terms and conditions of the Creative Commons Attribution (CC BY) license.

 https://doi.org/10.3390/cells15020122

Supplementary Materials: The following supporting information can be downloaded at: https://www.mdpi.com/article/doi/s1, .Table S1. Nucleotide sequences from the target miRNAs used in the present study. Table S2. Demographic and clinical characteristics of the study population according to cardiac disease (*n* = 64). Table S3. Demographic and clinical characteristics of the study population selected for miRNA discovery (*n* = 32). Table S4. Adipose tissue-related miRNAs with potential impact on diabetes and cardiac disease. Table S5. Linear mixed-methods model estimating the effects of tissue type, anthropometric characteristics, and CAD status on miRNA expression (*n* = 64). Table S6. Summary of miRNA expression changes according to CAD status in EAT and SAT. Table S7. Specific target gene symbols for each tissue. Table S8. Predicted gene ontology analysis (biological processes, BPs) pathways specific to each tissue. Gene ontology pathways. Table S9. Linear mixed-methods model estimating the effects of tissue type, anthropometric characteristics, disease status, and sex on miRNA expression (*n* = 64). Figure S1. Schematical representation of patient recruitment and group stratification according to the presence or absence of diabetes and cardiac disease. EAT, epicardial adipose tissue; CAD, coronary disease group; DM; diabetes mellitus group; NCAD, non-coronary disease group; NDM, non-diabetes mellitus group; SAT, subcutaneous adipose tissue. Figure S2. Schematical representation of the epicardial and subcutaneous adipose biopsies used for RNA pool. EAT, epicardial adipose tissue; CAD, coronary disease group; DM; diabetes mellitus group; NCAD, non-coronary disease group; NDM, non-diabetes mellitus group; SAT, subcutaneous adipose tissue. Figure S3. Heatmap of the most relevant miRNAs expressed in EAT and SAT. EAT, epicardial adipose tissue; DM; diabetes mellitus group; NDM, non-diabetes mellitus group; SAT, subcutaneous adipose tissue. Figure S4. MiRNAs expression levels in EAT and SAT regarding the presence or absence of DM. (a) miR-151a-5p, (b) miR-455-5p, and (c) miR-485-3p. *p*-values were determined using a two-way ANOVA after logarithmic (log_2) transformation. Data are presented as means ± SEMs. EAT, epicardial adipose tissue; DM; diabetes mellitus group; NDM, non-diabetes mellitus group; SAT, subcutaneous adipose tissue. Figure S5. Adipose tissue miRNA levels are influenced by tissue type under the same cardiac disease. (a) miR-93a-3p p, (b) miR-223-3p, (c) miR-324-5p. *p*-values were determined using a two-way ANOVA after logarithmic (log_2) transformation. Data are presented as means ± SEMs. * $p \leq 0.05$; *** $p \leq 0.0001$. EAT, epicardial adipose tissue; CAD, coronary disease group; NCAD, non-coronary disease group; SAT, subcutaneous adipose tissue. Figure S6. miRNA expression levels in EAT and SAT according to cardiac disease. (a) miR-155-5p, (b) miR-151a-5p, (c) miR-455-5p, and (d) miR-485-3p. *p*-values were determined using a two-way ANOVA after logarithmic (log_2) transformation. Data are presented as means ± SEMs. EAT, epicardial adipose tissue; CAD, coronary disease group; NCAD, non-coronary disease group; SAT, subcutaneous adipose tissue.

https://doi.org/10.3390/cells15020122

Supplementary Materials

Table S1. Nucleotide sequences from the target miRNAs used in the present study.

Primer Name	Primer type	Primer Sequences 5′ – 3′
**hsa-miR-151a-5p**	RT	CTCAACTGGTGTCGTGGAGTCGGCAATTCAGTTGAGACTAGACT
	FW	ACACTCCAGCTGGGTCGAGGAGCTCACA
**hsa-miR-93-3p**	RT	CTCAACTGGTGTCGTGGAGTCGGCAATTCAGTTGAGCGGGAAGT
	FW	ACACTCCAGCTGGGACTGCTGAGCTAGCA
**hsa-miR-223-3p**	RT	CTCAACTGGTGTCGTGGAGTCGGCAATTCAGTTGAGTGGGGTAT
	FW	ACACTCCAGCTGGGTGTCAGTTTGTCAAAT
**hsa-miR-155-5p**	RT	CTCAACTGGTGTCGTGGAGTCGGCAATTCAGTTGAG AACCCCTA
	FW	ACACTCCAGCTGGGTTAATGCTAATCGTGAT
**hsa-miR-886-3p**	RT	CTCAACTGGTGTCGTGGAGTCGGCAATTCAGTTGAGAAGGGTCA
	FW	ACACTCCAGCTGGGCGCGGGTGCTTACT
**hsa-miR-455-5p**	RT	CTCAACTGGTGTCGTGGAGTCGGCAATTCAGTTGAGCGATGTAG
	FW	ACACTCCAGCTGGGTATGTGCCTTTGGAC
**hsa-miR-485-3p**	RT	CTCAACTGGTGTCGTGGAGTCGGCAATTCAGTTGAGAGAGAGGA
	FW	ACACTCCAGCTGGGGTCATACACGGCTCT
**hsa-miR-324-5p**	RT	CTCAACTGGTGTCGTGGAGTCGGCAATTCAGTTGAGCACCAATG
	FW	ACACTCCAGCTGGGCGCATCCCCTAGGGC
**U6 snRNA**	RT	AACGCTTCACGAATTTGCGT
	FW	CTCGCTTCGGCAGCACA
**C.el-39**	RT	CTCAACTGGTGTCGTGGAGTCGGCAATTCAGTTGAGCAAGCTGA
	FW	ACACTCCAGCTGGGTCACCGGGTGTAAATC

RT, reverse primer; FW, forward primer. miRNA sequences were purchased from TAG Copenhagen A/S, Copenhagen, Denmark

https://doi.org/10.3390/cells15020122

Table S2. Demographic and clinical characteristics of the study population according to cardiac disease ($n = 64$).

	**NCAD**	**CAD**	***p*-Value**
N	38	26	
Male (M)	18 (47%)	17 (65%)	0.16
Age (years)	71.8 ± 1.8	69.0 ± 1.5	0.27
Cardiovascular risk factors			
Diabetes Mellitus	10 (26%)	8 (31%)	0.70
Hypertension	30 (79%)	20 (77%)	0.85
Dyslipidemia	26 (68%)	23 (88%)	0.06
Smoking	6 (26%)	9 (35%)	0.08
BMI	27.70 ± 0.58	26.46 ± 0.52	0.14
Family history of heart disease	0 (0%)	2 (8%)	0.11
Medication			
Antiplatelet	9 (24%)	14 (54%)	0.05
Antiarrhythmic	6 (26%)	2 (8%)	0.19
Anticoagulant	7 (18%)	6 (23%)	0.99
Insulin	2 (5%)	4 (15%)	0.30
Oral antidiabetic			
DPP4 inhibitor	0 (0%)	1 (4%)	0.27
DPP4 inhibitor + Biguanide	3 (8%)	0 (0%)	0.10
Sulfonylurea	1 (3%)	0 (0%)	0.35
Diuretic	16 (42%)	11 (42%)	0.42
ACEI	10 (26%)	11 (52%)	0.44
ARB	7 (18%)	10 (38%)	0.15
β blocker	9 (24%)	13 (50%)	**0.01**
Calcium channel blocker	1 (3%)	7 (27%)	**0.01**
Electrolyte – KCl	0 (0%)	2 (8%)	0.12
Statins	21 (55%)	18 (31%)	0.97
NCAD patients (n = 38)	**NDM**	**DM**	***p*-Value**
N	28	10	
Male (M)	13 (46%)	5 (50%)	0.85
Age (years)	71.4 ± 2.3	72.9 ± 2.5	0.72
BMI	27.7 ± 0.7	27.6 ± 1.0	0.91
Dysfunctional valve			
Aortic	21 (75%)	6 (60%)	0.37
Mitral	2 (7%)	0 (0%)	0.9
Tricuspid	-	-	-
Bivalvular	5 (18%)	3 (30%)	0.33
CAD patients (n = 26)	**NDM**	**DM**	***p*-Value**
N	18	8	
Male (M)	12 (67%)	5 (63%)	0.84
Age (years)	68.0 ± 1.9	71.4 ± 2.1	0.30
BMI	26.6 ± 0.7	26.2 ± 0.8	0.76
Dysfunctional valve			
Aortic	6 (33%)	2 (25%)	0.61
Mitral	1 (6%)	0 (0%)	0.48
Tricuspid	-	-	-
Bivalvular	1 (6%)	1 (13%)	0.43
Number of revascularized cardiac vessels			

https://doi.org/10.3390/cells15020122

1 vessel disease	5 (28%)	3 (38%)	0.84
2 vessels disease	8 (44%)	3 (38%)	0.47
3 vessels disease	2 (11%)	2 (25%)	0.48

Quantitative measurements (BMI and age) are presented as means ± SEM and an unpaired Student's t-test was performed. Categorical variables are reported as n (%) and compared using a Chi square test was applied. Significant *p*-values ($p \leq 0.05$) are highlighted in bold. ACEI, angiotensin-converting enzyme inhibitor; ARB, angiotensin II receptor blockers; BMI, body mass index; DM; diabetes mellitus group; DPP-4, dipeptidyl peptidase-4; NDM, non-diabetes mellitus group.

Table S3. Demographic and clinical characteristics of the study population selected for miRNA discovery (*n* = 32).

	**NDM**	**DM**	***p*-Value**
N	16	16	
Male (M)	9 (56%)	10 (63%)	0.72
Age (years)	74.6 ± 1.5	73.3 ± 1.3	0.49
Cardiovascular risk factors			
Hypertension	13 (81%)	16 (100%)	0.07
Dyslipidemia	12 (75%)	12 (75%)	>0.99
Smoking	3 (19%)	4 (25%)	0.67
BMI	26.56 ± 0.48	26.35 ± 0.49	0.76
Family history of heart disease	1 (6%)	1 (6%)	0.96
Medication			
Antiplatelet	6 (38%)	9 (56%)	0.36
Antiarrhythmic	2 (13%)	4 (25%)	0.41
Anticoagulant	3	5(31%)	0.47
Insulin	0 (38%)	6 (38%)	**0.008**
Oral antidiabetic			
Biguanide	0 (38%)	8 (50%)	**0.001**
DPP4 inhibitor	0 (38%)	1 (6%)	0.33
DPP4 inhibitor + Biguanide	0 (38%)	2 (13%)	0.16
Sulfonylurea	0 (38%)	1 (6%)	0.33
Diuretic	6 (38%)	12 (75%)	**0.039**
ACEI	6 (38%)	7 (44%)	0.84
ARB	5 (31%)	5 (31%)	0.78
β blocker	5 (31%)	9 (56%)	0.19
Calcium channel blocker	2 (13%)	2 (13%)	0.94
Electrolyte – KCl	1 (6%)	1 (6%)	0.96
Statins	10 (63%)	11 (69%)	0.91

Quantitative measurements (BMI and age) are presented as means ± SEM and an unpaired Student's t-test was performed. Categorical variables are reported as n (%) and compared using a Chi square test was applied. Significant *p*-values ($p \leq 0.05$) are highlighted in bold. ACEI, angiotensin-converting enzyme inhibitor; ARB, angiotensin II receptor blockers; BMI, body mass index; DM; diabetes mellitus group; DPP-4, dipeptidyl peptidase-4; NDM, non-diabetes mellitus group.

Table S4. Adipose tissue-related miRNAs with potential impact on diabetes and cardiac disease.

miRNA	Described in	Biological Role	References
miR-155-5p	CVD	Inflammation	[22]
miR-93-3p	Obesity	Brown adipogenesis in EAT; Obesity; Insulin resistance	[29] [30] [31]
	CVD	CVD biomarker	[32] [33] [34]
miR-223-3p	DM	Glucose uptake; Insulin resistance; Inflammation	[23] [24]
	CVD	CAD biomarker; Atherosclerosis; Inflammation;	[25] [26] [27] [28]
miR-324-5p	DM and Metabolic syndrome	Lipid accumulation; Metabolic dysfunction; Adiposity	[35] [36] [37] [38]
miR-485-3p	CVD	CAD biomarker Atherosclerosis Brown adipogenesis in EAT	[41] [42] [43]
miR-455-5p	CAD and DM	Brown adipogenesis in EAT	[4] [44] [45]
miR-151a-5p	CVD and Obesity	Obesity; Metabolic dysfunction; CAD biomarker	[39] [40]

CAD; coronary artery disease; CVD, cardiovascular disease; DM, diabetes mellitus; EAT, Epicardial adipose tissue.

https://doi.org/10.3390/cells15020122

Table S5. Linear mixed-methods model estimating the effects of tissue type, anthropometric characteristics, and CAD status on miRNA expression (n = 64).

Term	miR-155-5p	miR-93a-3p	miR-223-3p	miR-324-5p	miR-151a-5p	miR-455-5p	miR-485-3p
Intercept	**-2.817 (0.932)*** **	-1.173 (0.732)	-0.940 (0.946)	**-1.841 (0.735)***	**-1.688 (0.773)***	**-2.052 (0.750)****	-0.837 (0.557)
CAD	0.233 (0.312)	0.031 (0.244)	-0.264 (0.363)	0.138 (0.226)	0.364 (0.240)	0.017 (0.250)	-0.074 (0.205)
Tissue	-0.586 (0.258)*	**0.876 (0.178)*****	**1.374 (0.321)*****	**0.555 (0.142)*****	**0.407 (0.145)****	0.255 (0.182)	0.011 (0.184)
Age	0.023 (0.013)	0.005 (0.010)	0.012 (0.013)	0.021 (0.010)*	0.018 (0.011)	0.017 (0.010)	0.002 (0.007)
Sex	0.008 (0.237)	0.045 (0.196)	0.245 (0.253)	0.105 (0.192)	0.162 (0.208)	-0.005 (0.201)	0.251 (0.144)
CAD: Tissue	0.177 (0.399)	0.178 (0.277)	0.144 (0.505)	-0.028 (0.219)	-0.175 (0.226)	0.129 (0.284)	0.082 (0.286)
CAD: EAT	-0.233 (0.312)	-0.031 (0.244)	0.264 (0.363)	-0.138 (0.226)	-0.364 (0.240)	-0.017 (0.250)	0.074 (0.205)
CAD: SAT	-0.410 (0.316)	-0.208 (0.243)	0.120 (0.358)	-0.110 (0.224)	-0.189 (0.240)	-0.146 (0.249)	-0.008 (0.205)

A linear mixed-methods model was created, and data are presented as estimate (SE). The upper section displays model estimates for each variable. The final two rows summarize the Tukey-adjusted comparisons evaluating the effect of CAD status within each tissue, adjusted for age and sex. Significant p-values ($p \leq 0.05$) are highlighted in bold, *p < 0.05. **p < 0.01 and ***p < 0.001. CAD, coronary artery disease; EAT, epicardial adipose tissue; SAT, epicardial adipose tissue.

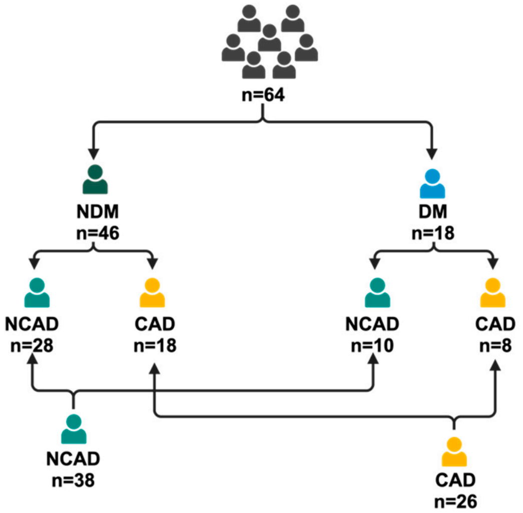

Figure S1. Schematical representation of patient recruitment and group stratification according to the presence or absence of diabetes and cardiac disease**.** EAT, epicardial adipose tissue; CAD, coronary disease group; DM; diabetes mellitus group; NCAD, non-coronary disease group; NDM, non-diabetes mellitus group; SAT, subcutaneous adipose tissue.

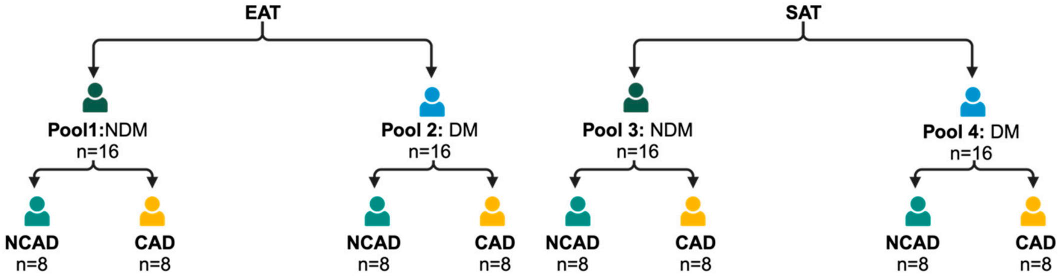

Figure S2. Schematical representation of the epicardial and subcutaneous adipose biopsies used for RNA pool. EAT, epicardial adipose tissue; CAD, coronary disease group; DM; diabetes mellitus group; NCAD, non-coronary disease group; NDM, non-diabetes mellitus group; SAT, subcutaneous adipose tissue.

https://doi.org/10.3390/cells15020122

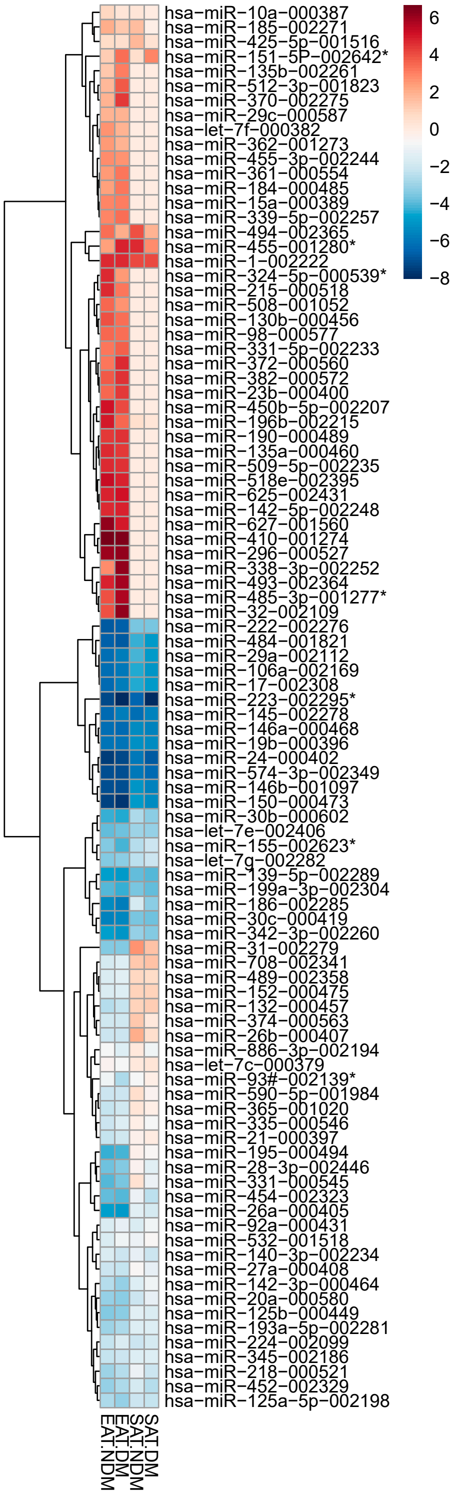

Figure S3. Heatmap from the most relevant miRNAs expressed in EAT and SAT. EAT, epicardial adipose tissue; DM; diabetes mellitus group; NDM, non-diabetes mellitus group; SAT, subcutaneous adipose tissue.

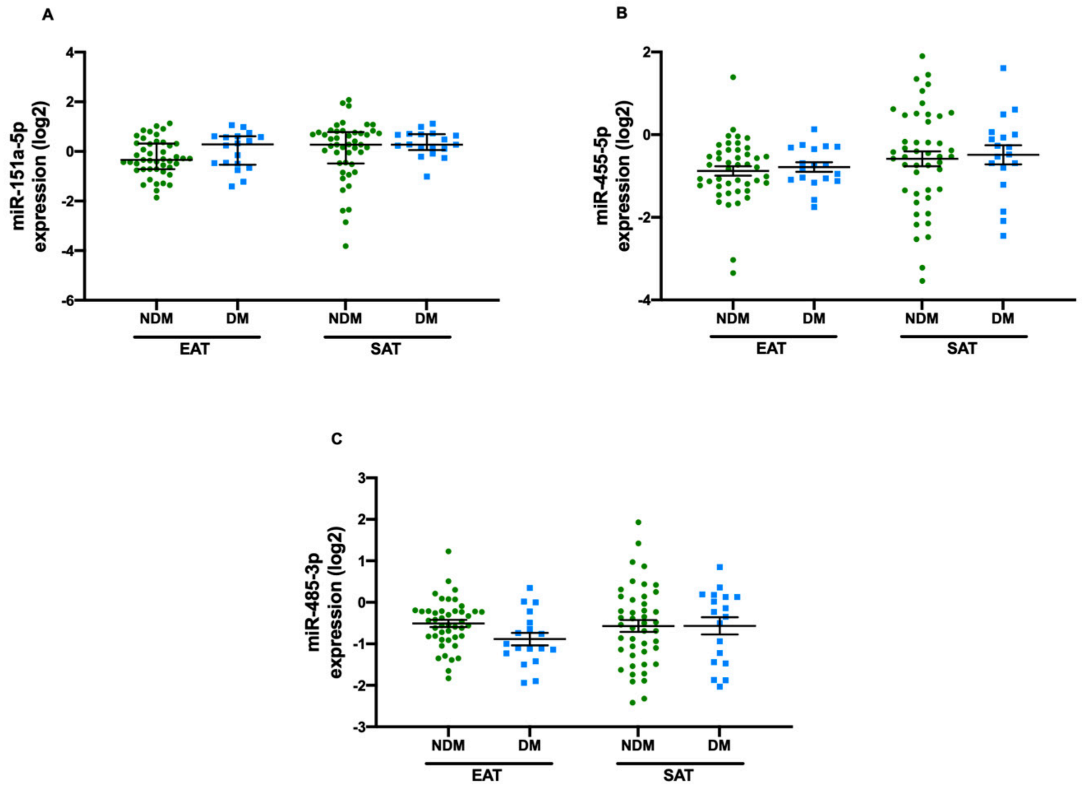

Figure S4. MiRNAs expression levels in EAT and SAT regarding the presence or absence of DM. **(A)** miR-151a-5p; **(B)** miR-455-5p; and **(C)** miR-485-3p. The *p*-values were determined using a two-way ANOVA after logarithmic (log2) transformation. Data are presented as mean ± SEM. EAT, epicardial adipose tissue; DM; diabetes mellitus group; NDM, non-diabetes mellitus group; SAT, subcutaneous adipose tissue.

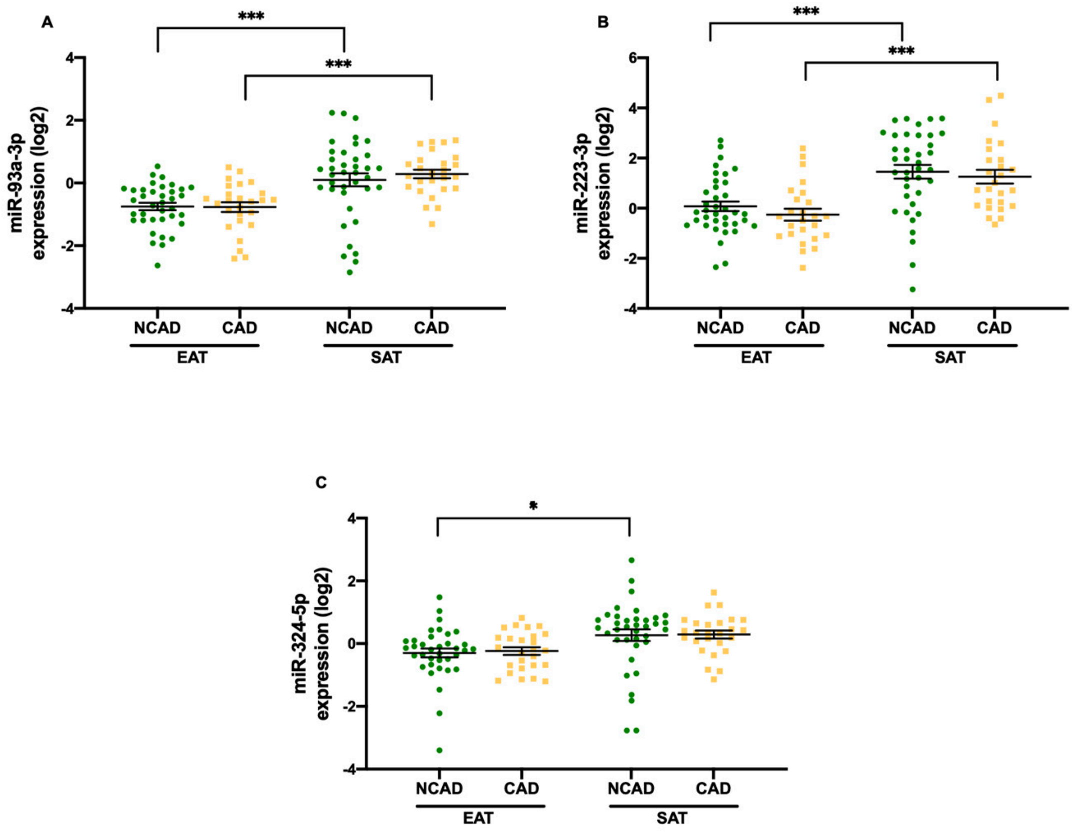

Figure S5. Adipose tissue miRNAs levels are influenced by the tissue under the same cardiac disease. (A) miR-93a-3p; **(B)** miR-223-3p; **(C)** miR-324-5p. The *p*-values were determined using a two-way ANOVA after logarithmic (log2) transformation. Data are presented as mean ± SEM. $^{*}p \leq 0.05$; $^{***}p \leq 0.0001$. EAT, epicardial adipose tissue; CAD, coronary disease group; NCAD, non-coronary disease group; SAT, subcutaneous adipose tissue.

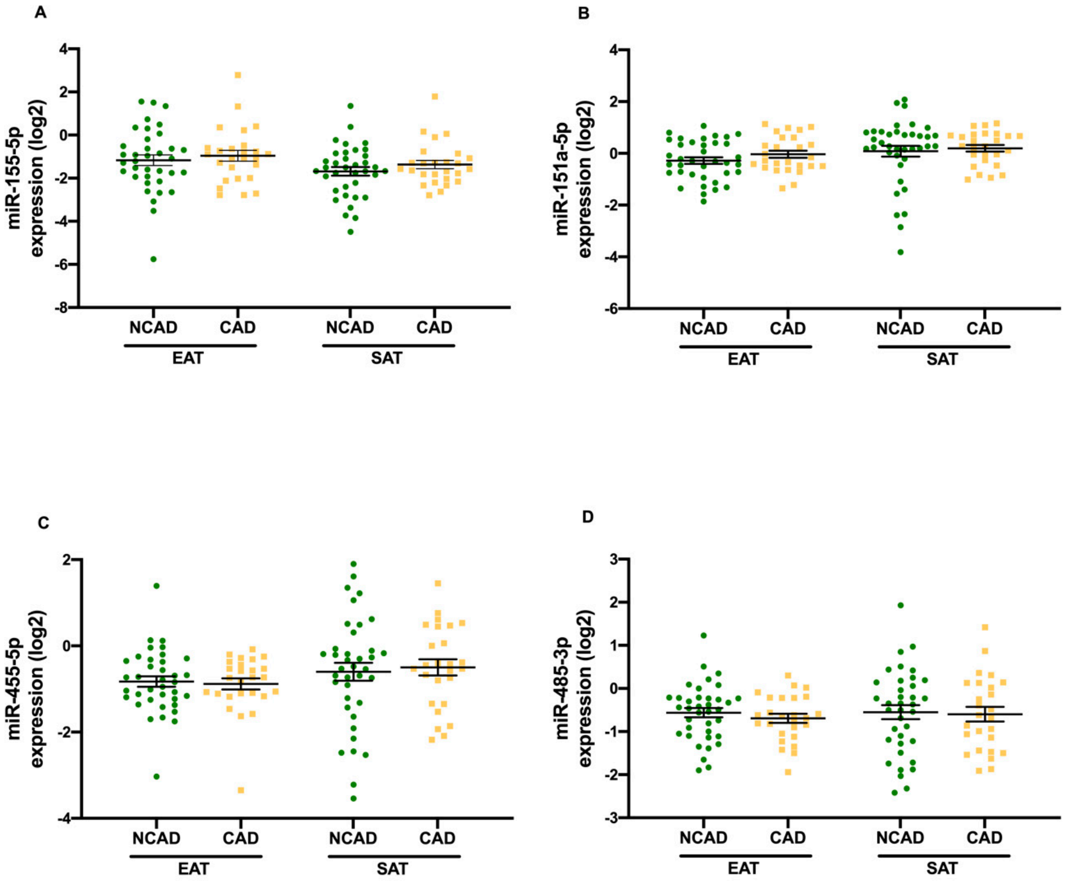

Figure S6. The miRNAs expression levels in EAT and SAT regarding the cardiac disease. (A) miR-155-5p; **(B)** miR-151a-5p; **(C)** miR-455-5p; and **(D)** miR-485-3p. The *p*-values were determined using a two-way ANOVA after logarithmic (log2) transformation. Data are presented as mean ± SEM. EAT, epicardial adipose tissue; CAD, coronary disease group; NCAD, non-coronary disease group; SAT, subcutaneous adipose tissue.

https://doi.org/10.3390/cells15020122

Table S6. Summary of miRNA expression changes according to CAD status in EAT and SAT.

	ANOVA		Mixed methods analysis		
**miRNA**	**Effect of Tissue**	**Effect of CAD**	**Effect of Tissue**	**Effect of CAD**	**Interaction (CAD:Tissue)**
miR-155-5p	–	–	+ associated with SAT ($p = 0.003$)	–	–
miR-93a-3p	NCAD: ↓ in EAT vs. SAT ($p = 0.007$)<br>CAD: ↓ in EAT vs. SAT ($p = 0.0004$)	–	+ associated with SAT ($p \leqslant 0.001$)	–	–
miR-223-3p	NCAD: ↓ in EAT v vs. SAT ($p = 0.002$)<br>CAD: ↓ in EAT vs. SAT ($p = 0.001$)	–	+ associated with SAT ($p \leqslant 0.001$)	–	–
miR-324-5p	NCAD: ↓ in EAT vs. SAT ($p = 0.03$)	–	+ associated with SAT ($p \leqslant 0.001$)	–	–
miR-151a-5p	–	–	+ associated with SAT ($p \leqslant 0.01$)	–	–
miR-455-5p	–	–	–	–	–
miR-485-3p	–	–	–	–	–

The *p*-values were determined using a two-way ANOVA after logarithmic (log_2) transformation. or by applying a linear mixed-methods model. *p* values were considered significant at $p \leqslant 0.05$. ↓, decreased by; ↓, increased by; +, positively; -, negatively; CAD, coronary artery disease group, EAT, epicardial adipose tissue; EAT, epicardial adipose tissue; NCAD, non-coronary artery disease group; SAT, epicardial adipose tissue.

https://doi.org/10.3390/cells15020122

Table S7. Specific target gene symbols for each tissue.

miRNA	Tissue	Targets genes
**miR-93-3p**	**EAT (n = 220)**	RPL17-C18orf32; TMEM151A; HOMEZ; LRRC6; HMGCS2; C12orf66; TRIM74; SLC26A1; SOD2; GADL1; SIGLEC8; GPR37L1; C10orf67; GPR55; RIPK4; TMEM63C; VAT1L; LMOD3; GOLGA6L10; FRMD5; IL24; PDZD3; TVP23A; ACTC1; WNT3; ARPC4-TTLL3; ULBP3; NPHS1; UGT2B4; SPRR2B; SMTNL2; FRMPD3; FAM83H; HAPLN4; CDHR1; FOXL1; BEX2; LRRC56; ANXA8; TRPA1; ESRP2; GALNT9; GPRIN2; MUC20; RNF43; AMIGO3; FGFR4; C22orf23; SLC9A3R2; OTUB2; BEND4; FAM104A; WNT16; FAM83A; HRNR; GSG1L; CLNK; MTUS2; FAM207A; FAM126A; CCDC103; SPIN2A; FAM153B; FAM184B; C1QTNF9B; SYNDIG1L; PCDH20; NDST3; LHX4; MUC6; MEX3A; PRIMA1; CXCR3; SNX32; POLR2F; PLA2G2D; PLEKHG4B; BCKDHA; CHRFAM7A; C16orf72; GRID2; C11orf45; MYCN; PHLDA2; NDUFA7; TFAP2A; GOLGA6L4; ZBTB8B; TSPEAR; SEC14L6; GPR17; PLGLB1; EVA1A; ZNF660; C20orf194; SOSTDC1; CTAGE8; FSBP; TMIE; NPY4R; DLK1; FUT2; TIMD4; EPHA10; RPRM; HAP1; WNT7B; PRR9; NXPH2; ZNF780A; CHRNA7; ZADH2; DERL3; SPIB; EHF; GAL3ST3; NUDT10; KIAA1522; ANXA13; TMEM159; DRAXIN; INSC; FAM102B; KIF18B; CELF6; KLHDC7B; DUOX2; MAL; SERPINB7; MPV17L; WT1; POC1B-GALNT4; TLL2; TIFAB; HSF2BP; CRB2; KLRK1; RSPO4; FAM167A; MORN3; ARHGEF39; ISY1-RAB43; LRRN4; FCRL2; CFC1; PRR15L; ZNF497; GBA; ABCG4; KY; SLC6A20; GREM1; BHLHB9; TNFRSF13C; BHLHA15; HMGA2; RNF165; CXCR5; PODNL1; C9orf64; UHRF1BP1; SGPP2; PACRG; DSC3; CNR2; MYH15; CRX; LRRC55; TOMM6; PCDH11Y; MSH5; PPAN-P2RY11; KCNH8; ADM2; MB; C16orf91; RAB3B; PLEKHN1; ACSM2A; CHRNA4; NKD2; SHISA9; ISM2; DNAJC28; FAM172A; GATA5; TCEAL5; ST6GAL2; TRIM39; SLC26A9; CHIA; ANGPTL6; ZNF280B; CHRM2; PRSS16; WFIKKN2; FAM189A1; DNASE1L2; AMN; CORO7; SEC14L4; LMX1B; DLGAP2; FAM160B2; CST2; WNT4; PTK6; DCC; PLA2G4B; WNT9B; XIRP2; AGMAT; NRG1; MARVELD2; CDH4; FAM160A1; NPFFR1; CENPM; GATA4; PRR3
	**SAT (n = 59)**	DCLK3; SYT13; GNAT1; OPRK1; IFITM10; SCN1A; DCX; SLC46A2; KCNH4; NSG2; PCDH11X; RPH3A; CAPN8; SLC25A2; MAF1; DKK3; OPCML; SLC22A13; CALN1; VWC2; UNC80; SHOX; GABBR2; AKR7L; NCAN; MAB21L3; ARL11; CREG2; CLDN2; CACNA1B; LSM10; CLVS2; PLD5; TAF1L; DIRAS2; SIT1; SLC24A2; FREM2; TRIM67; LRRTM4; CNIH2; ELAVL4; AADACL3; CACNG8; VNN1; FUT9; ST8SIA3; HOXA9; ARL3; CHD5; LSM1; KSR2; SP5; ERN1; GAD1; GPR39; SH2D5; LRRD1; MYBPHL
**miR-155-5p**	**EAT (n = 15)**	KIF26B; UHRF1BP1L; EHF; FAM126A; SOX10; SCG2; ACTA1; RAB3B; FGF9; IL17RB; OTUB2; TMEM200C; FAM104A; TBX20; MYB
	**SAT (n = 11)**	STXBP5L; C5orf64; ELAVL4; SHOX; PLD5; ECT2L; FUT9; ERN1; GABRA1; HECW1; SCN1A
**miR-223-3p**	**EAT (n = 4)**	GTSF1; FAM160B1; ABCG4; TNNI3K
	**SAT (n = 5)**	OPCML; ST8SIA3; SLC24A2; CTSV; SCN1A;
**miR-324-5p**	**EAT (n = 4)**	ISM2; C20orf194; ST6GAL2; AMHR2
	**SAT (n = 3)**	NCAN; DCX; CAMKV

EAT, epicardial adipose tissue; SAT, subcutaneous adipose tissue.

Table S8. Predicted gene ontology analysis (biological processes, BPs) pathways specific to each tissue. Gene ontology pathways

Tissue	Predicted pathways
**EAT (n = 49)**	mesonephros development (GO:0001823); regulation of muscle cell differentiation (GO:0051147); canonical Wnt signaling pathway (GO:0060070); response to growth factor (GO:0070848); immunoglobulin production (GO:0002377); regulation of cell projection organization (GO:0031344); B cell mediated immunity (GO:0019724); growth (GO:0040007); cellular process (GO:0009987); regulation of developmental growth (GO:0048638); response to extracellular stimulus (GO:0009991); positive regulation of Wnt signaling pathway (GO:0030177); regulation of cellular response to growth factor stimulus (GO:0090287); regulation of postsynapse organization (GO:0099175); mammary gland development (GO:0030879); circulatory system development (GO:0072359); negative regulation of cellular response to growth factor stimulus (GO:0090288); positive regulation of heart growth (GO:0060421); cell surface receptor signaling pathway (GO:0007166); regulation of neurotransmitter transport (GO:0051588); regulation of cell development (GO:0060284); mesenchyme development (GO:0060485); response to nutrient levels (GO:0031667); cell-cell signaling by wnt (GO:0198738); kidney development (GO:0001822); mesenchymal cell differentiation (GO:0048762); anatomical structure formation involved in morphogenesis (GO:0048646); regulation of membrane potential (GO:0042391); developmental growth (GO:0048589); mammary gland epithelium development (GO:0061180); macromolecule modification (GO:0043412); regulation of plasma membrane bounded cell projection organization (GO:0120035); regulation of heart growth (GO:0060420); digestive tract development (GO:0048565); cell-cell signaling (GO:0007267); production of molecular mediator of immune response (GO:0002440); Wnt signaling pathway (GO:0016055); generation of precursor metabolites and energy (GO:0006091); positive regulation of cell differentiation (GO:0045597); regulation of metanephric nephron tubule epithelial cell differentiation (GO:0072307); positive regulation of cardiac muscle tissue growth (GO:0055023); immunoglobulin mediated immune response (GO:0016064); negative regulation of response to stimulus (GO:0048585); positive regulation of cytoplasmic mRNA processing body assembly (GO:0010606); regulation of synaptic plasticity (GO:0048167); neuron projection morphogenesis (GO:0048812); negative regulation of transmembrane receptor protein serine/threonine kinase signaling pathway (GO:0090101); humoral immune response (GO:0006959); morphogenesis of an epithelium (GO:0002009)
**SAT (n = 2)**	cellular respiration (GO:0045333); regulation of molecular function (GO:0065009)

EAT, epicardial adipose tissue; SAT, subcutaneous adipose tissue.

https://doi.org/10.3390/cells15020122

Table S9. Linear mixed-methods model estimating the effects of tissue type, anthropometric characteristics, disease status, and sex on miRNA expression (*n* = 64).

**Term**	**miR-155-5p**	**miR-93a-3p**	**miR-223-3p**	**miR-324-5p**	**miR-151a-5p**	**miR-455-5p**	**miR-485-3p**
Intercept	-2.795 (0.918)	-1.165 (0.736)	-0.797 (0.921)	-1.779 (0.731)	-1.580 (0.774)	-2.019 (0.760)	-0.738 (0.548)
Tissue	-0.531 (0.267)	0.900 (0.183)	1.267 (0.325)	0.450 (0.144)	0.238 (0.149)	0.204 (0.186)	-0.202 (0.188)
Sex	-0.008 (0.304)	-0.011 (0.241)	0.062 (0.347)	0.004 (0.221)	0.054 (0.237)	-0.125 (0.248)	-0.021 (0.198)
Age	0.021 (0.013)	0.004 (0.010)	0.008 (0.012)	0.020 (0.010)	0.017 (0.011)	0.016 (0.010)	0.003 (0.007)
CAD	0.297 (0.238)	0.109 (0.201)	-0.221 (0.250)	0.112 (0.196)	0.263 (0.212)	0.080 (0.208)	-0.024 (0.145)
DM	0.467 (0.253)	0.205 (0.216)	0.764 (0.270)	0.259 (0.208)	0.249 (0.227)	0.070 (0.223)	-0.185 (0.154)
Tissue: sex	0.039 (0.395)	0.111 (0.274)	0.353 (0.483)	0.206 (0.216)	0.219 (0.223)	0.235 (0.278)	0.538 (0.278)
Sex: EAT	0.008 (0.304)	0.011 (0.241)	-0.062 (0.347)	-0.004 (0.221)	-0.054 (0.237)	0.125 (0.248)	0.021 (0.198)
Sex: SAT	-0.031 (0.306)	-0.100 (0.239)	-0.415 (0.342)	-0.210 (0.218)	-0.273 (0.235)	-0.110 (0.246)	**-0.517 (0.198) ****

A linear mixed method model was created, and data are presented as estimate (SE). The upper section displays model estimates for each variable. The final two rows summarize the Tukey-adjusted comparisons evaluating the effect of Sex within each tissue, adjusted for age. Significant *p*-values ($p \leq 0.05$) are highlighted in bold, **p* < 0.05. ***p* < 0.01 and ****p* < 0.001. DM, diabetes mellitus group; EAT, epicardial adipose tissue; SAT, epicardial adipose tissue.

https://doi.org/10.3390/cells15020122
